# Supplementary material for: ReaDDy 2: Fast and flexible software framework for interacting-particle reaction dynamics
Source: PLoS Comput Biol. 2019 Feb 28;15(2):e1006830. doi: 10.1371/journal.pcbi.1006830 (PMC6413953; doi:10.1371/journal.pcbi.1006830)
Supplement: S1 Table — This table contains parameters for the Debye-Hückel system in the results section. (PDF) [file pcbi.1006830.s001.pdf]

S1 Table. Parameters of density-dependent reaction kinetics.

Quantity	Symbol	Value	Unit
Thermal energy	k_BT	2.49	$\mathrm{kJ\,mol^{-1}}$
Volume	V	100^3	$\mathrm{nm^3}$
Radius A	r_A	1	nm
Radius B	r_B	0.8	nm
Radius C	r_C	1	nm
Diffusion coeff. A	D_A	0.01	$\mathrm{nm^2\,ns^{-1}}$
Diffusion coeff. B	D_B	0.0125	$\mathrm{nm^2\,ns^{-1}}$
Diffusion coeff. C	D_C	0.01	$\mathrm{nm^2\,ns^{-1}}$
Charge A	q_A	1.3	–
Charge B	q_B	-1	–
Charge C	q_C	0	–
Screening parameter	κ	3.82	$\mathrm{nm^{-1}}$
Debye-Hückel prefactor	$e^2\varepsilon_0^{-1}\varepsilon_r^{-1}$	2349	$\mathrm{kJ\,nm\,mol^{-1}}$
Repulsion energy	U_r	1.	$\mathrm{kJ\,mol^{-1}}$
Cutoff radius	r_{cutoff}	4.7	nm
Reaction radius	R	2.	nm
Equilibrium constant	K_{dilute}	6.16×10^{-5}	$\mathrm{nm^{-3}}$
Macroscopic rate constant	k_{on}	0.11	$\mathrm{nm^3\,ns^{-1}}$
Macroscopic rate constant	k_{off}	6.58×10^{-6}	$\mathrm{ns^{-1}}$
Microscopic rate constant	λ_{on}	5.61×10^{-3}	$\mathrm{ns^{-1}}$
Microscopic rate constant	λ_{off}	6.58×10^{-6}	$\mathrm{ns^{-1}}$
Timestep	τ	0.1	ns

This table contains parameters for the Debye-Hückel system in the results section.
